# Retrospective Multicenter Study of Human Granulocytic Anaplasmosis, France, 2012–2024

**DOI:** 10.3201/eid3112.250946

**Published:** 2025-12

**Authors:** Victor Gerber, Thomas Lemmet, Thomas Bonijoly, Baptiste Hoellinger, Anne Pachart, Amandine Woerly, Dominique De Briel, Emilie Talagrand-Reboul, Martin Martinot, Pierre Boyer, Yves Hansmann

**Affiliations:** Centre Hospitalier Universitaire de Strasbourg, Strasbourg, France (V. Gerber, T. Lemmet, B. Hoellinger, A. Woerly, E. Talagrand-Reboul, P. Boyer, Y. Hansmann); Hôpitaux Civils de Colmar, Colmar, France (V. Gerber, T. Lemmet, A. Pachart, D. De Briel, M. Martinot); Centre National de Référence des Borrelia, Strasbourg (V. Gerber, E. Talagrand-Reboul, P. Boyer, Y. Hansmann); Université de Strasbourg, Strasbourg (V. Gerber, E. Talagrand-Reboul, P. Boyer, Y. Hansmann); Centre Hospitalier de Sélestat, Sélestat, France (T. Bonijoly); Groupe Hospitalier de la Région de Mulhouse Sud-Alsace, Mulhouse, France (B. Hoellinger)

**Keywords:** Anaplasma phagocytophilum, vector-borne infections, bacteria, zoonoses, human granulocytic anaplasmosis, tick bite, cytopenia, secondary hemophagocytic lymphohistiocytosis, France

## Abstract

Human granulocytic anaplasmosis (HGA), caused by *Anaplasma phagocytophilum* transmitted through tick bites, remains poorly documented in France. We conducted a retrospective, multicenter study of cases in Alsace during 2012–2024, including 39 HGA episodes in 38 patients PCR positive for *A. phagocytophilum*. Most (63.2%) patients were men, median age was 60.5 years, and 76.3% lived in rural areas. A tick bite was reported in 61.6% of cases. Frequent symptoms included fever (97.4%), fatigue (61.5%), and headache (61.5%). Laboratory findings showed elevated C-reactive protein (100%), thrombocytopenia (94.9%), leukopenia (59.0%), and cytolysis (66.7%). One patient had secondary hemophagocytic lymphohistiocytosis. Most (87.2%) patients were hospitalized; none required intensive care unit admission. Doxycycline was administered in 29 cases, and all patients recovered. HGA should be considered in febrile patients with recent tick exposure and cytopenia. Although often benign, rare severe HGA forms can occur and justify increased clinical awareness, especially in *A. phagocytophilum*–endemic areas.

Human granulocytic anaplasmosis (HGA) is a tickborne zoonosis caused by *Anaplasma phagocytophilum*, an obligate gram-negative intracellular bacterium. *A. phagocytophilum* belongs to the order Rickettsiales, family Anaplasmataceae, genus *Anaplasma*, after its separation from the genus *Ehrlichia* (*1*). For decades, the bacterium was known to be associated with equine and ruminant disease, but in 1994, HGA was described in 6 patients in the United States (*2*). 

Clinical manifestations of HGA include febrile illness occurring 7–14 days after a tick bite along with mild and nonspecific symptoms, including malaise, headaches, myalgia, arthralgia, and vomiting (*3*). Rarely, severe and life-threatening manifestations, including pneumonia, hemophagocytosis, septic shock, respiratory distress syndrome, and death, can occur (*3*–*6*). Although uncommon, neurologic manifestations also can occur in HGA, including meningitis, encephalitis, stroke-like symptoms, or actual ischemic and hemorrhagic strokes, and represent a major cause of death among affected patients (*7*). Fatal HGA cases after blood transfusion have also been described (*8*). A combination of cytopenia, mostly thrombocytopenia, and elevated liver enzymes are the most frequent abnormal laboratory findings (*3*,*9*). Diagnostic confirmation is made on the basis of blood PCR, serology, blood smear observation, and culture testing (*3*,*10*). PCR appears to be best-suited diagnostic test and has a sensitivity of 74% and a specificity of 100% for *A. phagocytophilum* (*10*). 

Most HGA cases have been reported from the United States, specifically to the Centers for Disease Control and Prevention through the National Notifiable Diseases Surveillance System (https://www.cdc.gov/nndss), but some cases have also occurred in Europe and Asia (*11*). During 2000–2016, the number of HGA cases in the United States increased, rising from 1.4 to 7.27 cases/1 million persons/year, and HGA became the second most frequent tickborne disease after Lyme disease in a few US states (*12**–**15*). A 2024 literature review (*3*) showed that 3,019 anaplasmosis cases have been reported globally, including 2,942 HGA cases, but individual patient data were described in <20% of those cases (*3*). 

In France, an HGA case was reported in 2003 in which the patient presented with atypical pneumonitis; since then, only a few cases had been described, mostly in the northeastern regions (*4*,*10*,*16*,*17*). Alsace is among the northeastern regions of France where HGA cases have been described and where *Ixodes ricinus* ticks are known to carry *A. phagocytophilum* (*16*,*18*). Thus, we aimed to describe all known cases in Alsace to clarify the epidemiology of HGA by focusing on clinical presentations, laboratory findings, and patient outcomes.

## Materials and Methods

### Study Design and Setting

We conducted a 12-year, descriptive, retrospective multicenter study of all *A. phagocytophilum* PCR tests performed in the bacteriology laboratory of Strasbourg University Hospital (Strasbourg, France). Patient samples were obtained during January 1, 2012–December 31, 2024, from 4 centers in Alsace: Strasbourg, Sélestat, Colmar, and Mulhouse.

### Case Definition and Inclusion Criteria

We included all patients with confirmed HGA, which we defined as an *A. phagocytophilum*–positive PCR test on blood. We only included cases diagnosed in a hospital setting because PCR testing was requested exclusively by hospital physicians for inpatients or patients seen in emergency departments or for hospital-based consultations.

### Molecular Testing Methods

During 2012–2022, a simplex real-time PCR test targeting the *msp2/p44* gene of *A. phagocytophilum* was used (*16*). Since 2022, the laboratory has used a biplex PCR for simultaneous detection of *Neoehrlichia mikurensis* and *A. phagocytophilum*, in which the *A. phagocytophilum* target is still the *msp2* gene (*19*).

### Data Collection and Ethics

The exclusion criteria were as follows: patients with a positive PCR test performed outside the 4 defined hospital centers, patients under legal protection, or patients objecting to the use of their data or for whom nonopposition to the use of their data could not be obtained. We collected the patients’ demographic data (age and sex), outdoor activities (forest activities, hunting, fishing, gardening, and hiking), existence of a tick bite, travel within the previous month, medical history, clinical symptoms, laboratory findings, intensive care unit (ICU) admission, complications (including cardiac complications, kidney and respiratory failure, septic shock, multiorgan failure, secondary hemophagocytic lymphohistiocytosis [HLH] with an HScore, bleeding, splenic rupture, and death), and outcomes (*20*). The study was approved by the Ethical Committee of Medicine Odontology and Pharmacy Faculties and Hospitals, University Hospital of Strasbourg (approval no. CE-2024-104).

## Results

### Study Population

During the study period, we noted 1,032 PCR analyses for *A. phagocytophilum*, among which 55 (5.3%) were positive, representing 47 patients. Nine patients met exclusion criteria. Thus, the analysis included a total of 38 patients, 1 of whom had 2 distinct *Anaplasma* spp. infections, occurring 11 months apart. Therefore, we analyzed a total of 39 HGA cases (Figure).

**Figure:**
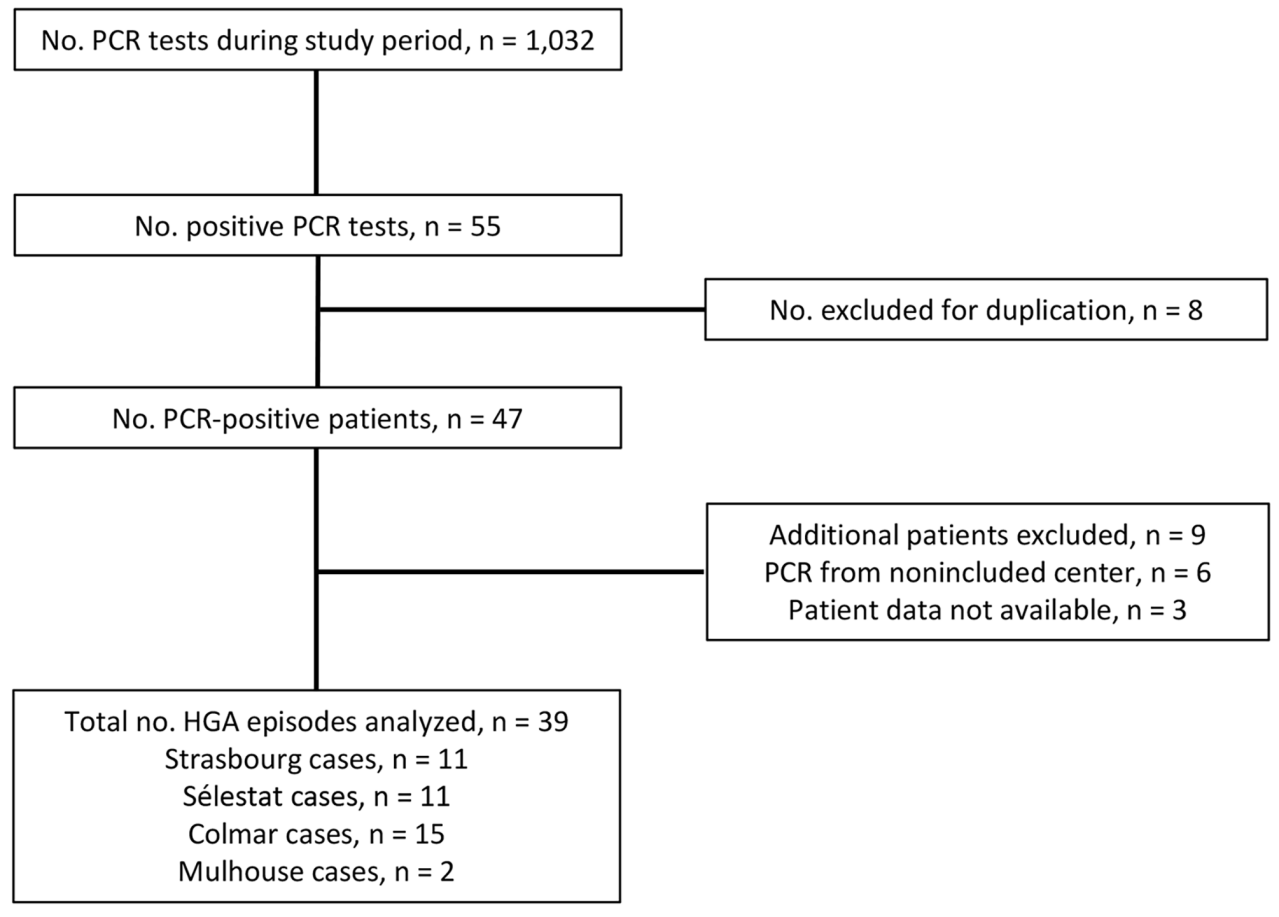
Flowchart of patient inclusion in a retrospective multicenter study of human granulocytic anaplasmosis, France, 2012–2024.

### Epidemiologic and Exposure Characteristics

The patients’ mean age was 60.5 years; 63.2% were men and 36.8% were women (Table 1). The most frequent underlying conditions were high blood pressure (34.2%), tobacco use (10.5%), and a history of immunosuppression (10.5%). No patient had liver disease or was pregnant at the time of diagnosis. Only 42.1% of patients engaged in outdoor activities, but most (76.3%) lived in rural areas (Table 1). 

**Table 1:** Characteristics and underlying conditions among 38 patients in a retrospective multicenter study of human granulocytic anaplasmosis, France, 2012–2024*

Patient characteristics	Value
Mean age, y (range)	60.2 (34–87)
Sex	
F	14 (36.8)
M	24 (63.2)
Participated in outdoor activities	16 (42.1)
Lived in a rural area	29 (76.3)
Underlying conditions	
High blood pressure	13 (34.2)
Tobacco use	4 (10.5)
Immunosuppression	4 (10.5)
Diabetes mellitus	2 (5.3)
Chronic kidney disease, GFR <60 mL/min	1 (2.6)
Chronic respiratory disease	1 (2.6)
Chronic cardiac failure, LVEF <45%	1 (2.6)

*Values are no. (%) except as indicated. One patient had 2 distinct *Anaplasma* spp. infections occurring 11 months apart. GFR, glomerular filtration rate; LVEF, left ventricular ejection fraction.

Among the 39 HGA cases, tick bite <30 days was reported in 61.5% (n = 24) (Table 2). The median time between tick bite and symptom onset was 7 (range 1–28) days. Only 1 patient traveled outside France, specifically in the United States, and 3 traveled in another region of France, 1 in Brittany and 2 in the south of France (Table 2).

**Table 2:** Clinical and epidemiologic characteristics of 39 cases in a retrospective multicenter study of human granulocytic anaplasmosis, France, 2012–2024*

Patient characteristics	Value
Tick bite <30 d	24 (61.5)
Median time between tick bite and symptom onset, d (range)	7 (1–28)
Travel history <30 d	
Outside France	1 (2.6)†
In France, outside the study region	3 (7.7)
Signs and symptoms	
Fever	38 (97.4)
Fatigue	24 (61.5)
Headache	24 (61.5)
Chills	15 (38.5)
Nausea	12 (30.8)
Vomiting	11 (28.2)
Abdominal pain	10 (25.6)
Myalgia	9 (23.1)
Anorexia	8 (20.5)
Cough	8 (20.5)
Other neurologic signs‡	5 (12.8)
Diarrhea	5 (12.8)
Hepatomegaly	4 (10.3)
Arthralgia	3 (7.7)
Confusion	3 (7.7)
Constipation	2 (5.1)
Splenomegaly	2 (5.1)
Rash	2 (5.1)
Pneumonia	1 (2.6)
Gravity signs	3 (7.7)
Hypotension	2 (5.1)
Hypoxia	1 (2.6)
No. patients with complications	13 (33.3)
Cardiac complications§	1 (2.6)
Pneumonitis	3 (7.7)
Acute kidney injury	7 (17.9)
Rhabdomyolysis	5 (12.8)
Confirmed secondary hemophagocytic lymphohistiocytosis on myelogram	1 (2.6)
Bleeding	1 (2.6)

*Values are no. (%) except as indicated. ARDS, acute respiratory distress syndrome. †Travel to Pennsylvania, USA. ‡Signs included dizziness, faintness, neuritis, or diadochokinesia. §Complications included cardiac arrhythmia and heart failure.

### Clinical Presentation and Complications

Among HGA cases, 97.4% (38/39) of patients exhibited fever, 61.5% (24/39) reported related headaches, 61.5% (24/39) reported fatigue, and 38.5% (15/39) reported chills. Of note, in 23 cases (59.0%), patients had >1 gastrointestinal symptom, including abdominal pain (25.6%, 10/39), nausea (30.8%, 12/39), vomiting (28.2%, 11/39), diarrhea (12.8%, 5/39), or anorexia (20.5%, 8/39). At the initial assessment, 3 patients had severe symptoms: 2 had hypotension and 1 had hypoxia. One third of patients had complications, the most frequent of which were acute kidney injury in 17.9% (7/39) of cases and rhabdomyolysis in 12.8% (5/39).

### Laboratory Findings and Microbiological Tests

The most frequent abnormal laboratory finding was cytopenia; 94.9% (37/39) of cases showed thrombocytopenia and 59.0% (23/39) showed leukopenia. Other irregular findings included elevated C-reactive protein (CRP) in all 39 cases and elevated liver enzyme levels in 26 (66.6%) cases (Table 3). Among case-patients, 8 had a myelogram performed, from which 1 secondary HLH case was confirmed, and 2 cases had an HScore >169, a threshold that has a sensitivity of 93% and a specificity of 86% for diagnosing secondary HLH. 

**Table 3:** Laboratory findings for 39 cases a retrospective multicenter study of human granulocytic anaplasmosis, France, 2012–2024*

Laboratory findings	Value	Reference range
Thrombocytopenia, no. (%)	37 (94.9)	
Median cells × 10^3^/μL (range)	59 (19–316)	150–400
Leucopenia, no. (%)	23 (59.0)	
Median cells × 10^3^/μL (range)	3.2 (1.02–13.01)	4.1–10.5
Anemia	8 (20.5)	
Median hemoglobin, g/dL (range)	13.4 (8.8–15.8)	13–18
No cytopenia, no. (%)	2 (5.1)	
Reactive lymphocytes, no. (%)	7 (17.9)	
Elevated liver enzymes, no. (%)	26 (66.6)	
Median AST, IU/L (range)	103 (24–509)	13–40
Median ALT, IU/L (range)	80.5 (27–829)	7–40
Elevated creatinine, no. (%)	7 (17.9)	
Median creatinine, μmol/L (range)	82 (43–233)	53–97
Elevated CRP	39 (100)	
Median CRP, mg/L (range)	130 (10–286)	0–5
Rhabdomyolysis, no. (%)	5 (12.8)	
Median CK, IU/l (range)	106 (40–1,012)	40–250
Myelogram performed, no. (%)	8 (20.5)	
Median HScore (range)†	76 (19–208)	NA
*Anaplasma phagocytophilum* testing, no. (%)		
Positive blood smear with morulae	8 (20.5)	NA
Positive IgM at diagnosis	9 (23.1)	Cutoff 1:20
Positive IgG at diagnosis	7 (17.9)	Cutoff 1:64
Other PCR positive, CSF	1 (2.6)	NA
Tickborne disease co-infection, Lyme disease‡	1 (2.6)	NA

*ALT, alanine aminotransferase; AST, aspartate aminotransferase; CRP, C-reactive protein; CK, creatine kinase; CSF, cerebrospinal fluid; NA, not applicable. †A score used for determining reactive hemophagocytic syndrome. ‡Co-infection data are based on medical records; no further diagnostic details available.

A positive blood smear was reported in 20.5% (8/39) of cases. *A. phagocytophilum* IgM and IgG indirect immunofluorescence assays were performed in 13 cases by using Focus Diagnostics *Anaplasma phagocytophilum* IFA Test Kit (Focus Diagnostics, https://www.focustechnologies.com) and applying a screening cutoff of 1:64 for IgG and 1:20 for IgM. Positive serum samples were subsequently titrated to determine the exact antibody titer. Nine cases were IgM positive and 7 were IgG positive. One patient had a positive *A. phagocytophilum* PCR result from a cerebrospinal fluid (CSF) sample. CSF analysis showed no leukocytes (0 cells/mL), 10 erythrocytes/mL, a protein level of 0.32 g/L (reference range 0.14–0.45 g/L), and glucose concentration of 0.71 g/L (within reference limits). In the absence of meningitis and because of mild blood contamination in the CSF, a false-positive PCR result was likely, although the patient did have neurologic symptoms, namely confusion.

### Treatment and Outcomes

Among the 39 HGA cases, most (87.2%, 34/39) patients were hospitalized, but none were admitted to the ICU (Table 4). Most (74.4%, 29/39 cases) patients were treated with doxycycline for a median duration of 7 (range 1–16) days. In 10 (25.6%) of 39 HGA episodes, patients did not receive doxycycline. Among those cases, 7 patients received no antimicrobial drug therapy, and the other 3 were treated with inappropriate antibiotics, including amoxicillin/clavulanic acid (n = 1), ceftriaxone (n = 1), and a sequential regimen of ceftriaxone and metronidazole followed by amoxicillin/clavulanic acid (n = 1); however, all patients recovered. Persistent asthenia at 3 months after diagnosis was reported in 3 cases.

**Table 4:** Characteristics and outcomes for 39 cases in a retrospective multicenter study of human granulocytic anaplasmosis, France, 2012–2024*

Patient characteristics	HGA episodes
Median delay between symptom onset and diagnosis, d (range)	8.5 (2–26)
No. hospitalized	34 (87.2)
Median hospitalization duration, d (range)	6 (1–13)
Intensive care unit admission	0
Antimicrobial treatment	
Ineffective therapy before doxycycline	13 (33.3)
No effective antibiotic therapy	10 (25.6)
Treatment with doxycycline	29 (74.4)
Median duration, d (range)	7 (1–16)
Outcomes	
Death <30 d after diagnosis	0
Recovered	39 (100)
Sequelae at 3 mo after diagnosis†	3 (7.7)

*Values are no. (%) except as indicated. HGA, human granulocytic anaplasmosis. †Including asthenia.

The patient who experienced 2 distinct infection episodes was not immunocompromised and received appropriate treatment with doxycycline for 14 days during the first episode. However, no serologic testing was performed during the first episode, preventing comparison of antibody titers between episodes.

## Discussion

In our study, we noted 39 cases of *A. phagocytophilum* infections occurring in 38 patients in 4 hospital centers in northeastern France over a 12-year period. The cases mostly occurred in patients who lived in rural areas and had few underlying conditions. Fever was the most frequent symptom and was often associated with other nonspecific manifestations, including asthenia, headaches, and digestive symptoms. Elevated CRP levels and thrombocytopenia were the most frequent abnormal laboratory findings and were observed in almost all cases. One third of patients had complications develop, including 1 case of HLH, but no patients required ICU admission. All patients recovered, even those who did not receive an appropriate antibiotic therapy.

Globally, most HGA cases have been described in the midwestern and northeastern parts of the United States, mainly in Minnesota, Wisconsin, and Rhode Island (*3*,*12**–**14*). Those cases were reported through a national surveillance system, and since 2000 a gradual increase in HGA incidence was noted, rising from 1.4 cases/1 million persons/year in 2000 to 7.27 cases/1 million persons/year in 2016 (*12**–**14*). In Europe, most HGA cases have been reported in Belgium, Poland, and Slovenia (*3*). However, relatively few confirmed HGA cases with clinical and laboratory findings have been reported, 156 cases from North America and only 46 from Europe. A 2024 systematic literature review reported only 6 published HGA case reports in France (*3*), and only 44 potential infections have ever been described in France, most in Alsace (*4*,*10*,*16*,*17*). Our study cases build on the reported HGA cases in France, and our investigation suggests that Alsace is an HGA hotspot, despite the low *Anaplasma* spp. prevalence rate among ticks (0.4% in nymphs and 1.2% in adults) (*18*,*21*). In addition, *A. phagocytophilum* has been detected in ticks elsewhere in France (*22*,*23*). The apparent absence of HGA cases might reflect regional variation in *A. phagocytophilum* ecotypes, and the human-pathogenic ecotype might be more prevalent in Alsace (*24*). Moreover, the regional difference diagnostic rates might be explained by higher awareness of HGA among clinicians in Alsace. 

Among case-patients, we found lower rates of underlying conditions and immunosuppression compared with previously published data (*3*,*9*). The most common suspected route of transmission is a tick bite, reported in up to 95% of confirmed HGA cases (*9*). In our study, a tick bite was reported for 61% (24/39) of cases, which is lower than the theoretical maximum, but remains notably higher than in several other studies on tickborne diseases, where most patients did not recall any tick exposure (*25*). That relatively high percentage might reflect greater awareness among persons in endemic rural areas or improved recognition of tick bites. One of the main risk factors for HGA and tick bite is outdoor activity, which we observed in only 42% (6/38) of our patients. The retrospective nature of the study probably underestimated that factor, especially considering that most patients lived in rural areas.

Blood transfusion is another route of transmission reported in the United States, but we had no cases from blood transfusion in our study (*3*,*9*). The lower incidence in our study cohort in comparison to the cohort from Europe and the systematic leukoreduction treatment of blood bags in France likely explain that difference. Infection though blood transfusion is much more concerning in immunocompromised patients, accounting for approximately half of the HGA cases in those patients (*3*,*9*). Other transmission routes include contact with human or animal body fluids or vertical infection; however, we found no such cases in our study. 

Almost all (97.4%, 38/39 cases) patients in our study had fever and thrombocytopenia (94.9%, 37/39 cases), and all had elevated CRP levels within 7 days after tick bite. The symptom frequency was higher in our study than in a previous report (*3*). In addition, of 38 patients in our study, 23 (59%) reported >1 gastrointestinal symptom, which is consistent with another literature review (*9*), highlighting the importance of suspecting HGA in endemic areas when gastrointestinal symptoms are associated with fever, thrombocytopenia, and elevated CRP levels (*9*). The frequencies of liver enzyme elevation (26/39 cases) and leukopenia (23/39 cases) in our study were similar to those of previous cohorts from Europe and North America. Although cytopenia and elevated liver enzyme levels commonly occur in HGA patients, a 2025 study reported that the triad of thrombopenia, leukopenia, and cytolysis occurred in only 23% of HGA cases, but that triad was associated with an increased risk for hospitalization (*26*).

The hospitalization rate for HGA in the United States is 31%, but our study reported an 87.2% hospitalization rate (*13*). That difference could be explained by our study design because we only enrolled hospital-diagnosed cases, whereas the US surveillance data also included milder cases. However, we did not observe many of the known hospitalization risk factors, including altered mental status, older age, underlying conditions, and immunosuppression (*26*), in our cohort. Among the 39 HGA episodes in our study, 13 (33.3%) cases had complications, which was a higher percentage than the complication rate reported in a previous study from Europe but less than that of the US study (*3*,*9*,*13*). That might reflect publication bias and differences in the definition of complication (*3*). Several studies have suggested that the US cases might have been more severe than the cases from Europe because more deaths and ICU transfers occurred in the US cohort (*3*,*9*,*28*). A 1996 study found that 3 (7%) of 41 patients required ICU admission (*27*). Data from US surveillance systems also found an HGA mortality rate of <1% (*12*,*13*). Although most HGA deaths have been reported in the United States, primarily in immunocompromised and older patients, 1 death has been described in Europe (*3*,*5*,*9*,*28*). 

Acute kidney injury was the most frequent complication in our cohort and is considered the most frequent complication among HGA cases, possibly in relation to rhabdomyolysis. We found 1 case of confirmed secondary HLH and 2 probable cases among patients with a high HScore (>169). Although rare, HLH is a well-described and potentially life-threatening complication of HGA; the reported HLH mortality rate is 23% among affected patients (*3*,*5*,*29*). HLH has also been increasingly associated with other tickborne infections, particularly *Ehrlichia* spp., *A. phagocytophilum*, and *Rickettsia* spp. infections (*30*). A 2024 review reported that *A. phagocytophilum* accounted for 12.2% of HLH cases linked to tickborne pathogens, following ehrlichiosis (45.9%) and rickettsioses (14.3%) (*30*). Of note, the mortality rate for HLH in that context appears lower than for HLH overall (16.3% vs. 41%), largely because of the availability of effective antimicrobial therapy when initiated promptly (*30*). Doxycycline remains the cornerstone treatment for HGA, but immunosuppressive agents could be considered in severe or refractory cases. In that 2024 review, 43.9% of patients received antimicrobial therapy alone, and 88.4% of those patients recovered without additional immunosuppression (*30*). Therefore, in endemic regions, *A. phagocytophilum* infection should be considered in patients with HLH of unclear origin.

Despite complications, including 1 severe case, none of the patients in our study died, even though only 74.4% received appropriate antibiotic therapy. Indeed, HGA can resolve spontaneously. Furthermore, a previous study showed that only 25% of HGA patients benefitted from antimicrobial drug therapy (*9*). Our study found a higher treatment rate, which was likely related to more severe HGA among our patients, although our patients had a shorter median treatment duration of 7 days compared with 12.9–14 days reported in previous literature (*3*,*9*).

Although retrospective studies are particularly useful for studying rare diseases, such as HGA, they have several limitations. First, our study design was limited by data completeness and might have a selection bias. Systematic HGA screening in febrile patients with tick exposure and cytopenia could improve the detection of the disease. A systematic search in our region during 2010–2012 and resulted in HGA diagnosis in 19 patients from 9 hospitals in Alsace (*10*). Furthermore, we chose to include only patients with a positive PCR test because PCR is the best diagnostic tool for *A. phagocytophilum* (*10*). However, some patients could have had true seroconversion because our study did not include that diagnostic confirmation criteria (*3*,*10*). Finally, a national surveillance system that includes the surveillance of all cases, particularly those with mild symptoms, would provide more precise epidemiologic data.

In conclusion, our study provides additional epidemiologic and clinical insights for HGA in Alsace, a region highly endemic for tickborne diseases. Although HGA is infrequent, it can lead to complications and is likely underdiagnosed. In the northeastern region of France where HGA is endemic, information campaigns targeting patients and healthcare workers could be beneficial. In addition, routine screening of patients with fever, leukopenia, thrombopenia, activation syndrome, or any combination of those signs and symptoms could also be useful.
